# Internal jugular vein thrombosis in a warfarinised patient: a case report

**DOI:** 10.1186/1752-1947-1-184

**Published:** 2007-12-20

**Authors:** Elizabeth Ball, Gareth Morris-Stiff, Mari Coxon, Michael H Lewis

**Affiliations:** 1 Department of Surgery, Royal Glamorgan Hospital, Ynysmaerdy, Llantrisant, Wales, UK

## Abstract

**Introduction:**

Internal jugular vein thrombosis (IJVT) is a rare but potentially fatal condition. It usually arises following trauma to the internal jugular vein but is also seen in association with coagulopathies and advanced malignancies as part of a para-neoplastic syndrome.

**Case presentation:**

We report a case of a 44 year old woman with a strong past medical history and family history of thrombotic disease who presented with abdominal pain and ascites. A stage III ovarian carcinoma was diagnosed and she underwent debulking of the tumour. She sustained a peri-operative haemorrhage and required insertion of a central line into the right internal jugular vein. At one month follow-up she presented as an emergency with a left neck mass and painful swallowing. A duplex ultrasound of her neck identified a left IJVT to the level of the brachiocephalic vein which had occurred despite warfarinisation and an INR of greater than 2. She was commenced on intravenous heparin and the swelling resolved over the course of a week.

**Conclusion:**

This case illustrates an unusual presentation of a rare condition. In this case, the precise aetiology is unclear as the IJVT may have been related to a coagulopathy or the presence of advanced malignancy and occurred despite adequate anticoagulation.

## Introduction

Internal jugular vein thrombosis (IJVT) was first described in 1912 by Long as a complication of a peritonsillar abscess [1]. It is an uncommon condition, but can be fatal. The two leading causes of IJVT are iatrogenic trauma secondary to jugular vein catheterisation, and repeated injections into the vein by intravenous drug users [2]. Other recognised causes include malignancy, ovarian hyperstimulation syndrome and coagulation disorders. The most serious complication from an IJVT is pulmonary embolism (PE). The aims of anticoagulation therapy, the treatment of IJVT, is to inhibit further thrombus formation and prevent embolisation.

## Case presentation

A 44 year old woman was admitted as an emergency on the surgical intake with right upper quadrant pain and vomiting. Her abdomen was distended and non-tender. She had a past history of a left-sided ileofemoral deep venous thrombosis (DVT) complicated by a PE, and a recurrent left-sided DVT following a long-haul flight. She had a positive family history of DVTs. The patient was known to be heterozygous for Factor V Leiden and Prothrombin 20210A variant. She was taking long-term warfarin, with an international normalised ratio (INR) maintained between 2.0 and 2.5.

An ultrasound scan showed a bulky left ovary and an ascitic tap contained malignant cells. Following resuscitation and investigation she was taken for laparotomy at which the pelvis was found to be 'frozen' with malignancy. There were secondary deposits throughout the peritoneum, omentum and liver. Biopsy of the left ovary provided a diagnosis of Stage III ovarian carcinoma. After debulking the tumour, the patient bled peri-operatively and was resuscitated on the intensive care unit, where a right-sided central jugular line and a left-sided peripheral line were placed. No lines were attempted or placed in the left neck. Her postoperative recovery was otherwise unremarkable and she was discharged after one week.

**Figure 1:**
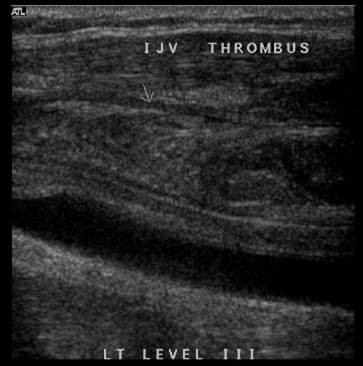
Duplex scan demonstrating an internal jugular vein thrombosis extending down to the brachiocephalic vein.

One month later the patient was readmitted with a two-day history of left-sided neck swelling and painful swallowing. Her INR on admission was 2.0. A duplex ultrasound of her neck identified a left IJVT to the level of the brachiocephalic vein (Figure 1). The patient was placed on intravenous heparin until her INR had stabilised at 3.0, when she was discharged. The swelling resolved over the course of a week and she had no recurrence of her symptoms.

## Discussion

Internal jugular vein thrombosis is an uncommon but potentially fatal condition. The most serious complications are septic emboli, septicaemia and pulmonary embolism, the latter having an incidence of 5% [1]. Cohen and colleagues recommend treating patients with one week of intravenous heparin and a three month course of oral warfarin therapy, together with a one week course of intravenous antibiotic prophylaxis.

In this case, the left IJVT thosmbosis would appear to have spontaneous. However, it si conceivable, that in addition to the recognised haematological risk factors, the insertion of a right-sided line at the time of cytoreduction surgery may have inadvertently traumatised the left brachiocephalic thus further adding to the risk of the subsequent left sided jugular thrombosis.

Carrington *et al. *[3] reported two cases of IJVT in patients with advanced malignancy, one from an ovarian cancer and the second a mesothelioma. Both were treated with heparin and warfarin. Metastatic adenocarcinoma induces a migratory thrombophlebitis secondary to the hypercoagulable state of cancer. This is seen in 5% of patients with cancer and more than 90% of patients with metastases will have some form of coagulation disorder [2].

Arullendran *et al. *[4] reported a case of a left IJVT in a patient with Factor V Leiden mutation. The importance of inactivated Factor V in haemostasis is that it inhibits clot formation. In a patient with the mutation, Factor V is resistant to inactivation, therefore coagulation is not inhibited and indeed there is a high risk of spontaneous thrombosis – a patient with a homozygous mutation has an eighty-fold increased risk of venous thrombosis. The presence of the prothrombin 20210A mutation also significantly increases the risk of venous thrombosis, and is the second most important risk factor for a DVT in the Caucasian population [5]. The recommended treatment of both conditions is long-term anticoagulation.

## Conclusion

This case has illustrated an unusual presentation of a rare condition and it remains uncertain as to the precise aetiology of the thrombosis. We would suggest that patients with IJVT should be formally anticoagulated with intravenous heparin and then be placed on oral anticoagulant therapy. For high risk patients such as those with factor V Leiden deficiency or the prothrombin 20210A mutation presenting with an IJVT, the INR should be maintained at a higher level, between 2.5 and 3.0, and consideration should be given to long-term warfarin therapy.

## Competing interests

The author(s) declare that they have no competing interests.

## Authors' contributions

MH Lewis and G Morris-Stiff were responsible for the concept, E Ball and M Coxon wrote the paper, and the manuscript was reviewed and edited by G Morris-Stiff and MH Lewis. All authors approved the final version.

## Consent

The authors confirm that written informed consent was obtained from the patient for publication of the manuscript.
